# Critical synchronization dynamics of the Kuramoto model on connectome and small world graphs

**DOI:** 10.1038/s41598-019-54769-9

**Published:** 2019-12-23

**Authors:** Géza Ódor, Jeffrey Kelling

**Affiliations:** 1 Institute of Technical Physics and Materials Science, Centre for Energy Research, P.O.Box 49, H-1525 Budapest, Hungary; 2 Department of Information Services and Computing, Helmholtz-Zentrum Dresden - Rossendorf, P.O.Box 51 01 19, 01314 Dresden, Germany

**Keywords:** Complex networks, Phase transitions and critical phenomena, Network topology

## Abstract

The hypothesis, that cortical dynamics operates near criticality also suggests, that it exhibits universal critical exponents which marks the Kuramoto equation, a fundamental model for synchronization, as a prime candidate for an underlying universal model. Here, we determined the synchronization behavior of this model by solving it numerically on a large, weighted human connectome network, containing 836733 nodes, in an assumed homeostatic state. Since this graph has a topological dimension *d* < 4, a real synchronization phase transition is not possible in the thermodynamic limit, still we could locate a transition between partially synchronized and desynchronized states. At this crossover point we observe power-law–tailed synchronization durations, with *τ*_*t*_ ≃ 1.2(1), away from experimental values for the brain. For comparison, on a large two-dimensional lattice, having additional random, long-range links, we obtain a mean-field value: *τ*_*t*_ ≃ 1.6(1). However, below the transition of the connectome we found global coupling control-parameter dependent exponents 1 < *τ*_*t*_ ≤ 2, overlapping with the range of human brain experiments. We also studied the effects of random flipping of a small portion of link weights, mimicking a network with inhibitory interactions, and found similar results. The control-parameter dependent exponent suggests extended dynamical criticality below the transition point.

## Introduction

Understanding the human brain, or in general neural systems is a great challenge of science, in particular the application of models and methods of statistical physics has been developing recently^1^. There are several types of whole brain models, ranging from continuous, integrate-and-fire models^2,3^ to discrete, activity spreading models^4,5^. All of them are effective ones, trying to describe different features of neural functions measurable by neuroscience experiments. While different versions of integrating fire models are more detailed and use larger parameter space, simple activity spreading models try to capture basic features, like the emergence of power-laws (PL) of quantities via critical behavior^6^. Following experiments, these are the neuron activity avalanche size and duration distribution tails, before finite size cutoff. Criticality in these systems can be defined by the diverging correlation volume, as we tune a control parameter to a threshold value.

Criticality is an attractive hypotheses, because information processing and dynamic range is optimal^7,8^. Neural activity avalanche measurements have found power-laws, which arise naturally close to a critical point of a phase transition^9–13^. The question of how a neural system would be tuned to this point has been debated. It was proposed to be by self-regulatory mechanisms^14^ leading to self-organized criticality^15^, or as the consequence of extended dynamical critical regions in spreading models^16,17^ in Griffiths Phases (GP)^18^. The measured scaling exponents have been found to be close to the mean-field transition values of discrete models^19^.

It is also known, that individual neurons emit periodic signals^20^, thus criticality may emerge by the collective behavior of oscillators at the phase synchronization transition point. However, not much is known about the dynamics of the synchronization or desynchronization process in these models^21–23^. Phase synchrony is essential for large-scale integration of information^24,25^, the role of the asynchronous state has remained more elusive^26^. Very recently theoretical analysis of the Ginzburg-Landau type equations arrived at the conclusion that empirically reported scale-invariant avalanches can possibly arise if the cortex is operated at the edge of a synchronization phase transition, where neuronal avalanches and incipient oscillations coexist^27^.

One of the most fundamental models, showing phase synchronization is the Kuramoto model of interacting oscillators^28^. This is defined on full graphs, corresponding to the mean-field (MF) behavior^29^, but as neural systems are not fully connected, we are interested in the phase synchronization transition in extended systems, where oscillators are located at graph points, possessing finite topological dimension *d*. This is defined by

1 $$\langle {N}_{r}\rangle  \sim {r}^{d},$$

where *N*_*r*_ is the number of node pairs that are at a topological (also called “chemical”) distance *r* from each other (i.e. a signal must traverse at least *r* edges to travel from one node to the other).

Phase transition in the Kuramoto model can happen only above the lower critical dimension *d*_*l*_ = 4^30^. Below *d*_*l*_ = 4 partial synchronization may emerge with a smooth crossover for strong coupling of oscillators, but a true, singular phase transition in the *N* → ∞ limit is not possible. On higher dimensional full or random graphs the Kuramoto equation exhibits universal scaling dynamics of the phase order parameter^31,32^. According to the theory of universality classes^19^ this “simple” model can describe other, more complex models of the brain. Very recently it has been studied analytically and computationally on a human connectome graph network of 998 nodes and in hierarchical modular networks (HMN), in which moduli exist within moduli in a nested way at various scales^33^. As the consequence of quenched, purely topological heterogeneity an intermediate phase, located between the standard synchronous and asynchronous phases was found, showing “frustrated synchronization”, metastability, and chimera-like states. This complex phase was investigated further in the presence of noise^34^ and on simplicial complex model of manifolds with finite and tunable spectral dimension^35^ as simple models for the brain.

Anatomical connections^36^ and the synchronization networks of cortical neurons^37^ indicate a small-world topology^38^. Here we will investigate the characteristic times, corresponding to synchronization or desynchroziation near the transition on a large human connectome graph *(KKI-18)* and compare it with results, obtained on 2d lattices with additional random, long range connections. The latter also exhibit small-world topology, because the 2d lattice has large clustering and the random, long range connections generate short path lengths among geometrically distant nodes. Our graphs are much larger than those considered before, allowing us to determine universal critical exponents that can be compared with experiments. Furthermore, we have heterogeneity in the intrinsic frequencies as well as in connection weights, which was found to be crucial in case of threshold model simulations^39^. Previously, extended discrete threshold model simulations of activity avalanches on *KKI-18* did not support a critical phase transition^39^. It turned out the weight heterogenities were too strong to allow the occurrence of criticality. This means that only the strongly connected hubs played a role in the activation/deactivation processes and weak nodes just followed them. As this appears to be unrealistic and uneconomic in a brain of billions of neurons, an input sensitivity equilibration was assumed via variable, node dependent thresholds. This makes the system homeostatic and simulations proved the occurrence of criticality, as well as robust Griffiths effects^39,40^ in spreading models. Indeed, there is some evidence that neurons have a certain adaptation to their input excitation levels^41^ and can be modeled by variable thresholds^42^. Very recently comparison of modeling and experiments arrived at a similar conclusion: equalized network sensitivity improves the predicting power of a model at criticality in agreement with the FMRI correlations^43^.

Even more naturally, homeostasis can be achieved in real brains via inhibitory neurons^44–48^, suppressing communications. This provides an alternative way for modifying the positive, undirected links of the *KKI-18* graph to test the phase synchronization of the Kuramoto model in the presence of random, negative couplings.

## Models and Methods

We consider the Kuramoto model of interacting oscillators^28^, with phases *θ*_*i*_(*t*) located at *N* nodes of networks, which evolve according to the dynamical equation

2 $${\dot{\theta }}_{i}(t)={\omega }_{i,0}+K\sum _{j}{W}_{ij}\,\sin \,[{\theta }_{j}(t)-{\theta }_{i}(t)]$$

Here, *ω*_*i*,0_ is the intrinsic frequency of the *i*-th oscillator, drawn from a Gaussian distribution with zero mean and unit variance and the summation is performed over other nodes, with connections described by the weighted adjacency matrix *W*_*ij*_. The global coupling *K* is the control parameter of this model, by which we can tune the system between asynchronous and synchronous states. We follow the properties of the phase transition through studying the Kuramoto order parameter defined by

3 $$R(t)=\frac{1}{N}|\mathop{\sum }\limits_{j=1}^{N}{e}^{i{\theta }_{j}(t)}|,$$

which is non-zero, above a critical coupling strength, *K* > *K*_*c*_ tends to zero for *K* < *K*_*c*_ as $R\propto \sqrt{1/N}$ or exhibits a growth at *K*_*c*_ as

4 $$R(t,N)={N}^{-1/2}{t}^{\eta }{f}_{\uparrow }(t/{N}^{\tilde{z}}),$$

in case of an incoherent initial state, with the dynamical exponents $\tilde{z}$ and *η*. In case of a coherent initial state it decays as:

5 $$R(t,N)={t}^{-\delta }{f}_{\downarrow }(t/{N}^{\tilde{z}}),$$

characterized by the dynamical exponent *δ*. Here *f*_↑_ and *f*_↓_ denote different scaling functions.

**Figure 1:**
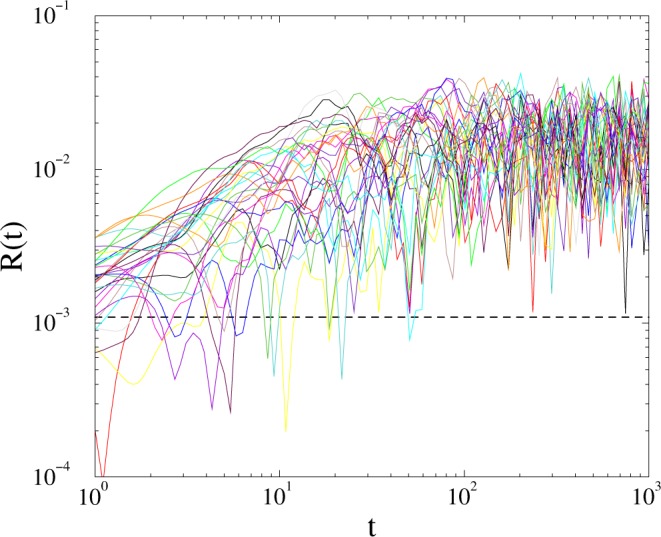
Evolution of *R*(*t*) for single realizations on the *KKI-18* graph at *K* = 1.7. The dashed line shows the threshold value $R=1/\sqrt{(N)}=0.001094$, where we measure the characteristic times: *t*_*x*_ of first cross.

We have also investigated the de-synchronization duration distributions by starting the system from fully synchronous or asynchronous states, near *K*_*c*_ by measuring the time *t*_*x*_ until *R*(*t*_*x*_) first fell below the threshold value: ${R}_{T}=1/\sqrt{N}$, related to the synchronization noise in the incoherent phase (see Fig. 1). For this measurement we performed ≃10^4^ runs, using independent random *ω*_*i*,0_ intrinsic frequencies and applied histogramming with increasing bin sizes: Δ*t*_*x*_ ∝ *t*_*x*_^1.12^ to estimate the probability distribution *p*(*t*_*x*_).

The following graphs have been considered:

1. 2d lattices with additional, random long-range connections such that 〈*k*〉 = 5 (2dll). We used periodic boundary conditions, simulating high dimensional graphs with supposedly mean-field behavior.
2. Weighted, symmetric large human connectome graph: *KKI-18*^49^ downloaded from the Openconnectome project^50^.
3. *KKI-18*, with 5% of the links turned to inhibitory: *KKI-18-I*

We applied the fourth order Runge-Kutta method from Numerical Recipes^51^ and the boost library odeint^52^ to solve Eq. (2) on various networks. Step sizes: Δ = 0.1, 0.01, 0.001 have been tested, but finally Δ = 0.1 precision found to be sufficient. Generally, the Δ < 0.1 precision did not improve the stability of the solutions, but caused smaller fluctuations due to the chaotic behavior of Eq. (2) which could be compensated by averages over many independent samples with different *ω*_*i*,0_. The criterion *ε* = 10^−12^ was used in the RK4 algorithm and we parallelized the RK4 for NVIDIA graphic cards (GPU), by which we could achieve a ~×40 increase in the throughput with respect to a single 12-core CPU. It is important to note, that first we verified the parallel GPU code, by comparing results on smaller sizes with those, obtained by the serial CPU program. Algorithmic and benchmark details will be discussed elsewhere^53^.

We measured the Kuramoto order parameter with a fixed *K*, by increasing the sampling time steps exponentially

6 $${t}_{k}=1+{1.08}^{k},$$

which is a common method in case of PL asymptotic time dependences. In practice we estimate *t*_*x*_ = (*t*_*k*_ + *t*_*k*−1_)/2, where *t*_*k*_ was the first measured crossing time. The initial conditions were generally *θ*_*i*_(0) ∈ (0, 2*π*] phases, with uniform distribution, describing fully disordered states. However, for comparison we also performed runs starting from the fully synchronized state: *θ*_*i*_(0) = 0. Probability distribution tails were fitted using the least squares fit method above thresholds, fixed by visual inspection of the results. To see the corrections to scaling we determined the effective exponents of *R* as the discretized, logarithmic derivative of Eq. (4) at these discrete timesteps *t*_*k*_, near the transition point

7 $${\eta }_{{\rm{eff}}}=\frac{\mathrm{ln}\,\langle R({t}_{k+3})\rangle -\,\mathrm{ln}\,\langle R({t}_{k})\rangle }{\mathrm{ln}({t}_{k+3})-\,\mathrm{ln}({t}_{k})}.$$

Here the brackets denote sample averaging over different initial conditions.

**Figure 2:**
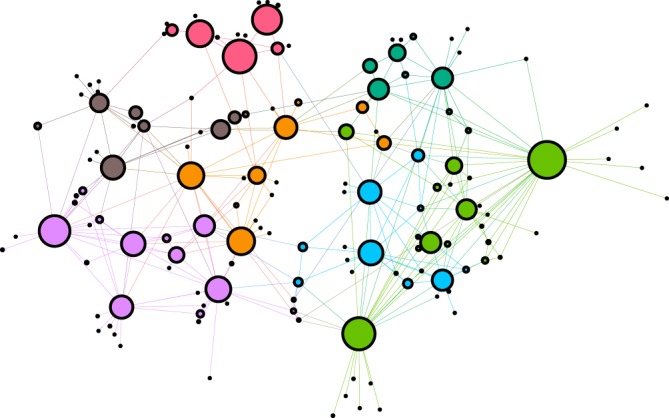
Network of the modules of the *KKI-18* human connectome graph. The size of circles is proportional with the number of nodes.

The *KKI-18* graph has been downloaded from the Open Connectome project repository^50^. This network was generated from Diffusion Tensor Imaging (DTI)^54^, approximating the *structural connectivity* of the white matter of a human brain. It comprises *N* = 836733 nodes, connected via 41523931 undirected edges, and several small sub-components, which were ignored here. Note, that results are not sensitive to removing the disconnected sub-components or deleting as much as 20% of the unidirectional links making up this graph. Solving (2) on this graph allows running extensive dynamical studies on present day CPU/GPU clusters, large enough to draw conclusions on the scaling behavior without very strong finite size effects. These connectomes of the human brain possess 1 mm^3^ resolution, using a combination of diffusion weighted, functional and structural magnetic resonance imaging scans. They are symmetric, weighted networks, where the weights measure the number of fiber tracts between nodes. The large graph “KKI-18” used here is generated by the MIGRAINE method, described in^55^. They exhibit hierarchical levels by construction from the Desikan cerebral regions with (at least) two quite different scales. The graph structure can be seen on the Fig. 2, where the modules were identified by the Louvain algorithm^56^, then the network of modules was generated and finally visualized using the Gephi tool^57^. This identified 144 modules, with sizes varying between 8 and 35202 nodes.

In^49^ it was found that, contrary to the small world network coefficients, these graphs exhibit topological dimension slightly above *D* = 3 and a certain amount of universality, supporting the selection of *KKI-18* as a representative of the large human connectomes available. This dimensionality suggests weak long-range connections, in addition to the *D* = 3 dimensional embedding and warrants to see heterogeneity effects in dynamical models defined on them.

To keep the local sustained activity requirement for the brain^4^ and provide a homeostatic state, we modified *KKI-18* by normalizing the incoming weights of node *i* in^39^: ${W^{\prime} }_{i,j}={W}_{i,j}/{\sum }_{j\in {\rm{neighb}}.{\rm{of}}i}{W}_{i,j}$ at the beginning of the simulations.

In addition, it is well known that excitation is balanced globally by the inhibitory cells, which is assumed to be a 20% fraction of all neurons. However, the vast majority of inhibitory connections are local as these cells have small dendritic and axonal trees^58^. Their range coincides roughly with the 1 *mm*^3^ of voxels in the connectivity data of *KKI-18*. The tracts, obtained by DTI are in contrast mostly excitatory axonal fibers; middle and long range connections are made by axons of pyramidal cells, which are excitatory. But these axons still could target excitatory and inhibitory cells in a voxel’s area. To model this, we flipped the signs of weights of 5% randomly selected links *i* as *W*″_*i*,*j*_ = −*W*′_*i*,*j*_, creating thus a modified graph called: *KKI-18-I*. Such links are against local synchronization and can be considered as an inhibition mechanism of possible information retrieval mechanism via resonance^59^.

## Results

### The 2dll graph

**Figure 3:**
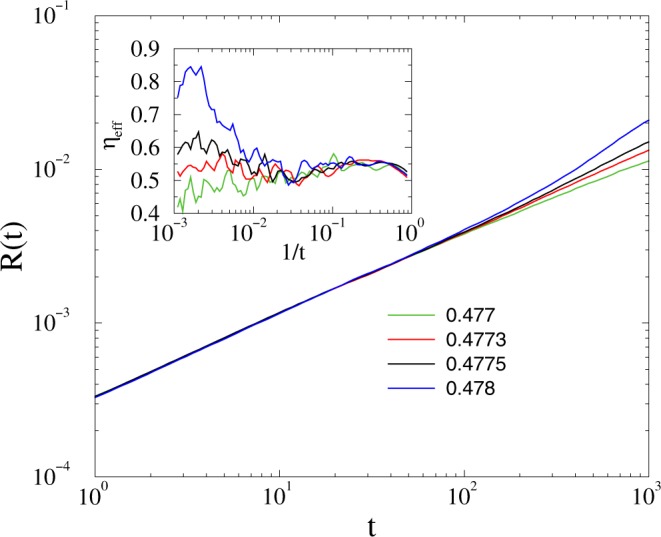
Growth of the average *R* on the 2dll model near the synchronization transition point for *K* = 0.477, 0.4773, 0.4775, 0.478 (bottom to top curves). Inset: the corresponding local slopes defined by (7).

First we studied the growth of *R*(*t*) on the 2dll model of linear size *L* = 6000 by starting from states of oscillators with fully random phases and by averaging over 5000–10000 *ω*_*i*,0_ realizations up to *t* = 10^3^. As Fig. 3 shows power-law growth of synchronization emerge up to *t* ≃ 100 in the coupling region: 0.477 ≤ *K* ≤ 0.478. Following that the *R*(*t*) curves veer up or down, depending on being super or sub-critical. Note, that for *t* ≳ 800 the curves begin to break down, owing to the finite size effect, when the growing correlation volume: $\xi \propto {t}^{\tilde{z}}$ exceeds the system size *N* = *L*^2^. Looking at the effective exponents defined by (7) one can estimate the critical point: *K*_*c*_ = 0.4775(3), as we expect in the asymptotic 1/*t* → ∞ limit constant local slopes. Off-critical cases exhibit up or down veering curvatures. Here one can read-off the asymptotic value: *η* = 0.55(10), on the local slope inset of Fig. 3, which is different from the MF value *η* = 0.75, expected for the Kuramoto model^31^ by scaling relations. The obvious discrepancy can be the consequence of very strong corrections-to scaling or some other quench disorder effect discussed further in^32^. Note, that in^31^, in case of fully connected graphs, the *η* = 0.75 exponent could hardly be seen, probably as the consequence of finite size and time limitations. While^31^ achieved sizes *N* ≤ 819200, here we provide results for much larger systems, containing *N* = 36000000 nodes, but without full topological order.

**Figure 4:**
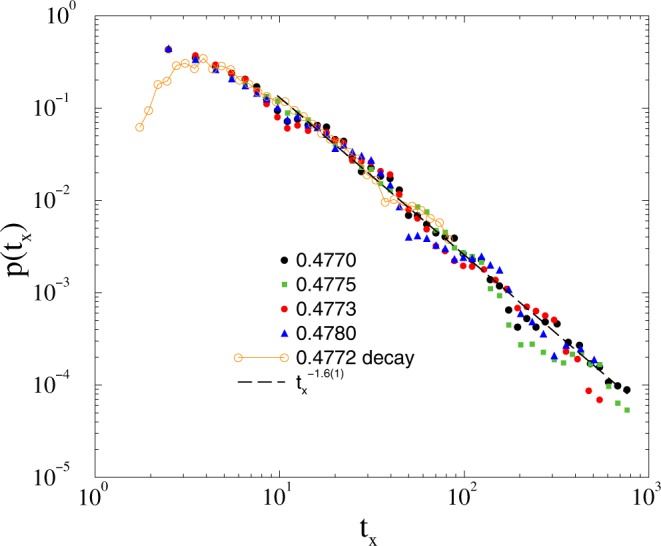
Duration distribution of *t*_*x*_ on the 2dll model for growth with *L* = 6000: *K* = 0.477 (bullets), 0.4773 (boxes), 0.4775 (diamonds), 0.478 (triangles) and decay with *L* = 1000 for *K* = 0.4772 (solid line). The dashed line shows a PL fit to the *K* = 0.477 line for the tail region: *t*_*x*_ > 10.

Instead of going into the details we just show that the relaxation time distributions, both for growth and for decay result in a robust fat tail behavior at the critical point, characterized by *τ*_*t*_ = 1.6(1) (see Fig. 4). The evolution of single realizations, in case of growth runs are shown on Fig. 1. The dashed line denotes the threshold at which the first passage time *t*_*x*_ is measured. We have also measured *t*_*x*_ in the case of fully coherent initial states in systems up to *t*_max_ = 10^4^. Figure 4 shows the tails of *p*(*t*_*x*_) around the critical point for incoherent initial conditions for *L* = 6000 and for coherent initial state with with *L* = 1000. In the latter case the decay occurs following a long transient time, thus the time is divided by a factor 100, but one can observe the same type of PL tails at *K* ≃ 0.477.

With this precision one cannot see a difference in the numerical scaling behavior at the critical point, but we note that the *τ*_*t*_ = 1.6(1) estimate is slightly above that one could obtain using the scaling relation

8 $${\tau }_{t}=1+\delta =1.5,$$

connecting dynamical exponents, see for example^19^. Note, that the *δ* = 0.6(1) result is in agreement with those of the extensive decay results, presented in^32^ and suggest that *δ* ≃ *η* on this scale, ruling out possible strong artifacts of the threshold value selection.

### The connectome graph

#### Normalized, positive weights

**Figure 5:**
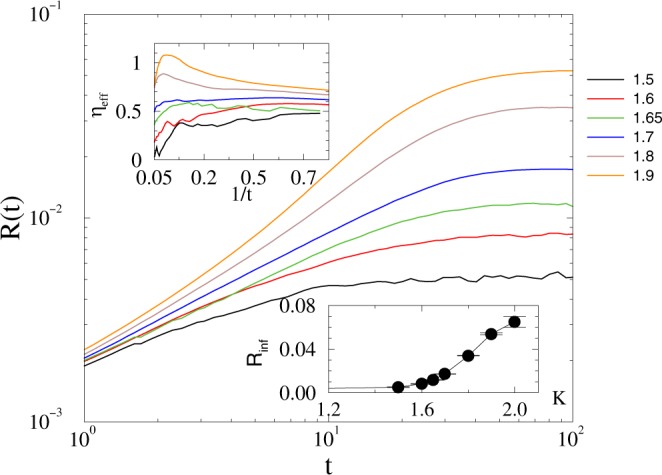
Growth of the average *R* on the *KKI-18* graph near the synchronization transition point for *K* = 1.5, 1.6, 1, 65, 1.7, 1.8, 1.9 (bottom to top curves). Upper left inset: Effective exponents, defined by (7) for the same data, down inset: steady state *R*(*t* → ∞) as the function of the global coupling.

In case of the *KKI-18* graph first we determined the crossover point via the inflexion condition, which separates up (convex) and down (concave) veering curves of the growth runs (see Fig. 5). As we can see, the transition is much smoother than what we obtained in the 2dll graph. The lower inset of Fig. 5) shows the steady state values *R*(*t* → ∞) as the function of *K*, with a very low level of synchronization above the transition. This smooth crossover behavior is not surprising, as the topological dimension of this graph is: *d* = 3.05 < *d*_*l*_ = 4^49^. This behavior is in agreement with PET and fMRI studies, which suggest that the magnitude of activity change from rest to task is rather small. Looking at the shapes of the *R*(*t*) curves and the saturation level of the corresponding local slopes we can estimate this crossover at: *K*_*c*_ = 1.65(5), with an effective scaling exponent *η*_eff_ ≃ 0.6(1). This exponent value is smaller than the *η* = 0.75 MF value for the Kuramoto model, but close to our 2dll graph results.

Having determined the transition point we run the numerical solver at control parameter values below *K*_*c*_, by starting with thousands of random initial states and measuring the first crossing times *t*_*x*_, when *R* fell below: $1/\sqrt{(}N)=0.001094$ (see Fig. 1).

**Figure 6:**
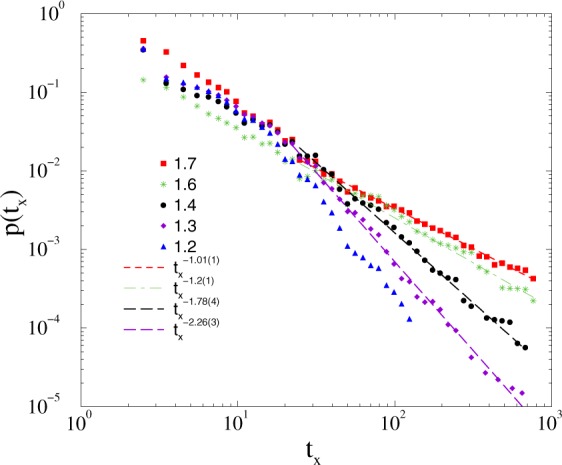
Duration distribution of *t*_*x*_ on the *KKI-18* model for growth *K* = 1.7 (boxes), 1.6 (stars), 1.4 (bullets), 1.3 (+), 1.2 (up triangles). The dashed lines shows PL fits for the tail region *t*_*x*_ > 20.

Following a histogramming procedure, with PL growing bin sizes in *t*_*x*_, we obtained the distributions *p*(*t*_*x*_), which exhibit PL tails, characterized by the exponents 1 < *τ*_*t*_ < 2 (see Fig. 6). Here the almost ~1/*t* decay at *K*_*c*_ ≥ 1.7 marks synchronized phase, with singular behavior; at *K*_*c*_ one obtains *τ*_*t*_ ≃ 1.2.

The *τ*_*t*_ = 1.2(1) is out of the range of neuro experiments: 1.5 < *τ*_*t*_ < 2.4^13^, but a good agreement/overlap can be found in the sub-threshold region. To investigate the effects of inhibition as in^39^ here we also studied the modified *KKI-18* graph, in which we flipped a small fraction of weight links randomly.

#### Inhibitory weights

**Figure 7:**
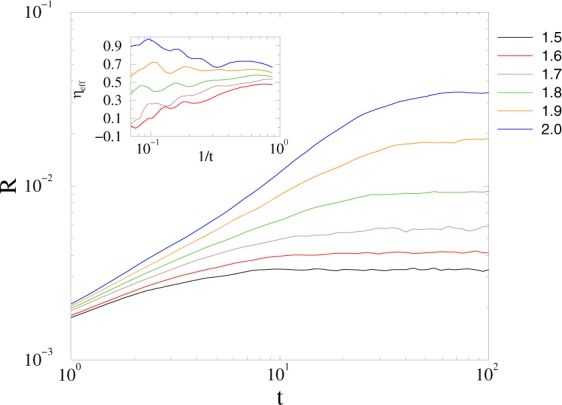
Growth of the average *R* on the inhibitory *KKI-18-I* graph near the synchronization transition point for: *K* = 1.5, 1.6, 1.7, 1.8, 1.9, 2.0 (bottom to top curves). Inset: effective exponents, defined by (7) for the same curves.

We repeated the analysis of the previous section for the *KKI-18-I* graph, possessing 5% inhibitory link fraction symmetrically: *W*″_*ij*_ = *W*″_*ji*_ = −*W*′_*ij*_. First we located the transition point as shown on Fig. 7.

**Figure 8:**
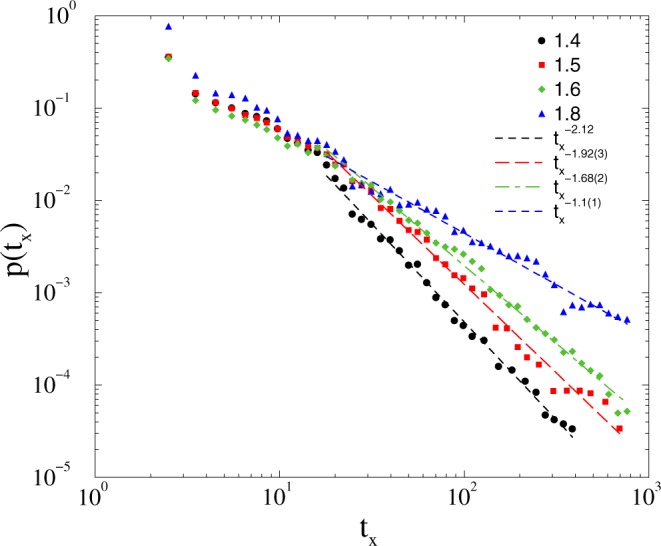
Duration distribution of *t*_*x*_ on the *KKI-18-I* model for growth *K* = 1.4 (bullets), 1.5 (boxes), 1.6 (diamonds), 1.8 (triangles). The dashed line shows PL fits to the tail region: *t*_*x*_ > 20.

The crossover to synchronization occurs at *K*_*c*_ = 1.9(1), slightly higher than in case of the *KKI-18* network. The tails of the *p*(*t*_*x*_) probability distributions exhibit PL-s with 1 < *τ*_*t*_ ≤ 2 in the 1.4 < *K* < 1.8 region. These exponent values overlap the range of experiments (see Fig. 8).

The large variation of *τ*_*t*_ below the transition, suggests the strong disorder may cause some GP effects, but in the lack of true phases we can only claim resemblance with recent results on power-grid networks^60^, where we pointed out a relation to a phenomena called frustrated synchronization^33,35^.

We have redone this analysis for another random 5% inhibitory link graph sample and arrived to the same results. In^39^ the robustness of GP in case of the threshold model dynamical behavior has been tested by the random neglection of 20% of links in one direction. Here we considered the situation of the neglection of all links in one direction: *W*″_*ij*_ = −*W*′_*ij*_, *W*″_*ji*_ = 0. Even in this extreme anisotropic case one case find a similar extended scaling region below a very smooth crossover as shown in the Supplementary Material.

**Figure 9:**
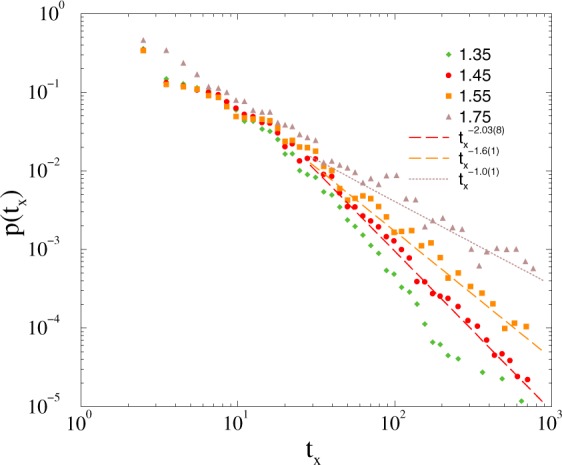
Duration distribution of *t*_*x*_ on the *KKI-18-I* model in case of 5% inhibitory node assumption for *K* = 1.35 (+), *K* = 1.45 (bullets), 1.55 (boxes), 1.75 (triangles). The dashed line shows PL fits to the tail region: *t*_*x*_ > 20.

Finally, we considered graphs with 5, 10, 20% inhibitory node assumptions, by flipping signs of (out or in) link weights of these randomly selected sites. Note, that nodes here represent big bunches of neurons. We show the duration distribution results here for one of the 5% inhibitory node case, when signs of out links are reversed. Below the synchronization transition point, which is at *K*_*c*_ = 1.7(1) we can find again a region: 1.35 < *K* < 1.7, where PL tailed de-synchronization duration durations emerge as before (see Fig. 9), characterized by the exponents 1 < *τ*_*t*_ < 2.

We have also studied the synchronization behavior on graphs with fully inhibited node cases of 5, 10, 20%, by flipping the weight signs of in-links. Even without weight normalization this produces crossovers at *K*_*c*_ ≃ 0.18 with *η*_eff_ ≃ 0.23(5), different from the *η* ≃ 0.6 value we found up to now. The tails of the *p*(*t*_*x*_) probability distributions exhibit PL-s with well inside the range of experiments (see Supplementary Material).

## Conclusion and Discussion

Brain experiments support evidence for power-law distributed activity avalanches. These have been explained mainly by discrete, threshold type of models, showing exponents close to the MF values, in agreement with neuro measurements. Oscillatory activity is widespread in dynamic neuronal networks^20^. The Blue brain project^61^ suggests that the cortical dynamics operates at the edge of a phase transition between an asynchronous phase and a synchronous one with emerging oscillations^62^. A recent MF theory showed the emergence of scale-free avalanches at the edge of synchronization^27^.

An extension, taking into account network heterogenities of a large human connectome is provided here, within the framework of the Kuramoto model. We determined the phase synchronization transition points and provided characteristic time exponents, which describe synchronization or de-synchronization events. We found good agreement with the neuro-experimental values in an extended range below the crossover point. We investigated the case, when 5, 10, 20% fraction of the randomly selected links or nodes are set inhibitory. The obtained *τ*_*t*_ duration exponents have been found to be invariant for these fractions and are in the range of experiments. This conclusion has already been derived within the framework of discrete threshold models^39,40^.

At the transition point the Kuramoto model on these homeostatic *KKI-18* network exhibits: *τ*_*t*_ = 1.2(1), well below the results for the 2dll graph, *τ*_*t*_ = 1.6(1), which is expected to be a system of MF interactions. However, even for this MF like model the dynamical exponents were found to be slightly away from the Kuramoto MF values^31^. This can be the result of enormous corrections to scaling, or due to quenched heterogeneity effects of the 2dll graph. The details of this problem is discussed in^32^. The effective growth exponent on the investigated connectome networks is *η* ≃ 0.6, near to the 2dll graph results, except for the node inhibited case, where *η*_eff_ ≃ 0.25(5) has been found.

Although in the *KKI-18* graph the topological dimension is below *d*_*l*_ = 4, a crossover behavior can clearly be identified. Around this smeared transition we found scale-free de-synchronization ‘avalanche’ tails, like in case of the dynamical criticality of GP, pointing out relation to possible frustrated synchronization effects^33,35^. Modules of the connectome graph enhance rare-region effects or frustrated synchroniztion domains.

As it was discussed in^39^ such coarse grained connectomes suffer possible sources of errors, like unknown noise in the data generation; underestimation of long connections; radial accuracy, influencing endpoints of the tracts and hierarchical levels of the cortical organization; or transverse accuracy, determining which cortical area is connected to another. Still important modifications, such as inhibitory links, directedness, or random loss of connections up to 20% confirmed the robustness of dynamical scaling, suggesting that fine network details may not play an important role. It is also important, that the PL tail in the weight distribution is similar to what was obtained by a synaptic learning algorithm in an artificial neural network^63^. A very recent experimental study has provided confirmation for the connectome generation used here^64^. Those results suggest that diffusion MRI tractography is a powerful tool for exploring the structural connectional architecture of the brain.

From a neuroscience point of view one may find the Kuramoto model too simplistic to describe the brain. However, at least in the weak coupling limit equivalence of phase-oscillator and integrate-and-fire models has been found^65^, that may hold for the sub-critical region, where we observed the dynamical scaling. At criticality universal scaling is expected to hold and thus the exponents obtained for this model can well describe the synchronization transition of other, more complex models. We believe that investigating a basic model is an important and necessary first step for neuroscience as this can provide a representative for a whole class of more real ones.

An interesting continuation of this work would be the study of the frequency entrainment of oscillators, which can exhibit real phase transition at *d* = 3.05 of the KKI-18 graph, or consideration of more complex models or graphs than the ones investigated here. Another open point could be the determination of avalanche sizes in these synchronization processes. The codes and the graphs used here are available on request from the corresponding author.

## Supplementary information

Supplementary information
