# Supplementary material for: Critical synchronization dynamics of the Kuramoto model on connectome and small world graphs
Source: Sci Rep. 2019 Dec 23;9:19621. doi: 10.1038/s41598-019-54769-9 (PMC6928153; doi:10.1038/s41598-019-54769-9)
Supplement: Supplementary file 1 — Supplementary information [file 41598_2019_54769_MOESM1_ESM.pdf]

Supplementary information
Critical synchronization dynamics of the Kuramoto model on connectome and small world graphs

Géza Ódor[1], Jeffrey Kelling[2*]

[1] *Institute for Technical Physics and Materials Science, Centre for Energy Research of the Hungarian Academy of Sciences, P.O.Box 49, H-1525 Budapest, Hungary*

[2] *Department of Information Services and Computing, Helmholtz-Zentrum Dresden - Rossendorf, P.O.Box 51 01 19, 01314 Dresden, Germany*

Here we show results for various realizations of the KKI-18-I graph to test universality. First we provide the case of the main text: 5% weight link flips, but for the fully asymmetric case: one-directional connections between nodes i and j. First we tried to estimate the transition point using the growth runs as shown on Figs S1.

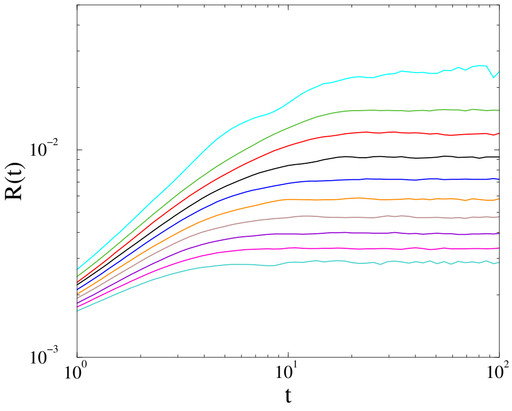

Supplementary Figure S 1: Growth of the average R on the inhibitory *KKI-18-I* graph near the synchronization transition point for: $K = 1.3$, 1.4, 1.5, 1.6, 1.7, 1.8, 1.9, 2.0, 2.1, 2.3 (bottom to top curves).

The crossover to synchronization seems to be much smoother as before, one cannot determine it using the inflexion point criterion. The tails of the $p(t_x)$ probability distributions exhibit PL-s with $1 < \tau_t \leq 2$ in the $1.3 < K < 2.1$ region. The $\tau_t = 1$ marks the transition point, because it means a singular probability distribution. These exponent values overlap the range of experiments (see Fig. S2).

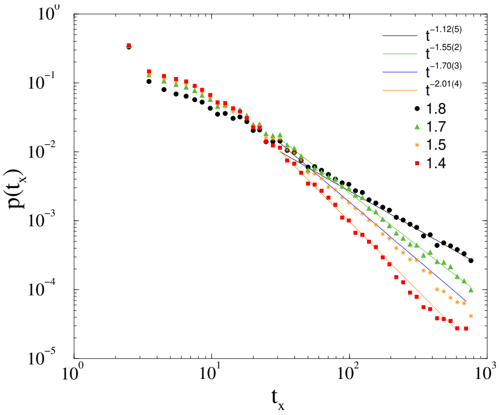

Supplementary Figure S 2: Duration distribution of t_x on the *KKI-18-I* model for growth $K = 1.4$ (boxes), 1.5 (stars), 1.7 (triangles), 1.8 (bullets). The dashed line shows a PL fits to the tail region: $t_x > 30$.

Next we show the results for the R growth at 5% inhibited nodes without weight normalization (Fig. S3) as well as for the duration distributions (Fig. S4). As one can see this produces crossover at $K_c \simeq 0.18$ with $\eta_{\text{eff}} \simeq 0.23(5)$, different from the $\eta \simeq 0.6$ value

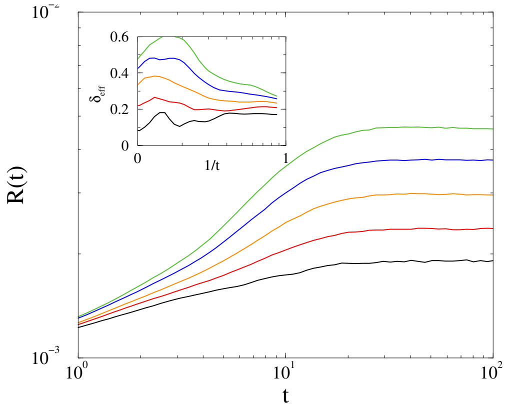

Supplementary Figure S 3: Growth of the average R on the *KKI-18* graph near the synchronization transition point for $K = 0.1$, 0.15, 0.17, 0.18, 0.19 (bottom to top curves) for 5% inhibited nodes. Inset: the corresponding local slopes).

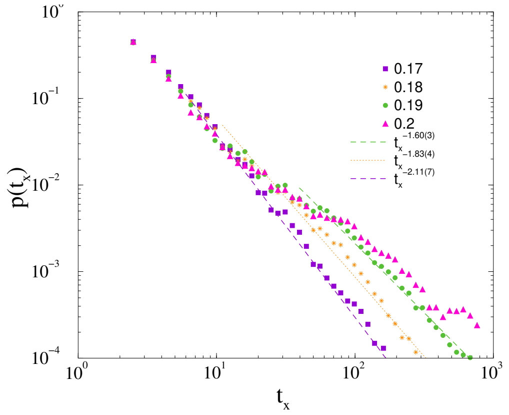

Supplementary Figure S 4: Duration distribution of t_x on the *KKI-18-I* model for growth $K = 0.17$ (boxes), 0.18 (stars), 0.19 (bullets), 0.20 (triangles), for 20% inhibited nodes. The dashed line shows a PL fits to the tail region: $t_x > 20$.

We have repeated this analysis for 10 and 20% node fractions, but basically we found the same results, except from a slight shift of the non-universal transition point location. The results are summarized in the Table I.

	5%	10%	20%
λ_c	0.17(1)	0.18(1)	0.20(1)
η	0.23(2)	0.23(2)	0.25(5)
τ_t	1.6-2.2	1.5-2.14	1.8-2.3

TABLE I: Summary of the synchronization transition results for the KKI-18 graph with different inhibited node fractions.
